# The Effect of Sex and Age on Bone Morphology and Strength in the Metacarpus and Humerus in Beef-Cross-Dairy Cattle

**DOI:** 10.3390/ani11030694

**Published:** 2021-03-05

**Authors:** Michaela Gibson, Rebecca Hickson, Penny Back, Keren Dittmer, Nicola Schreurs, Chris Rogers

**Affiliations:** 1 School of Agriculture and Environment, Massey University, Private Bag 11-222, 4442 Palmerston North, New Zealand; R.Hickson@massey.ac.nz (R.H.); P.J.Back@massey.ac.nz (P.B.); N.M.Schreurs@massey.ac.nz (N.S.); C.W.Rogers@massey.ac.nz (C.R.); 2 School of Veterinary Sciences, Massey University, Private Bag 11-222, 4442 Palmerston North, New Zealand; K.E.Dittmer@massey.ac.nz

**Keywords:** bone strength, fracture, humerus, metacarpal, sex

## Abstract

**Simple Summary:**

Bone strength in long bones is dependent on the strain it is exposed to via the forces from locomotion and bodyweight. The two strategies to increase bone strength (and reduce strain) are increasing either bone size or bone density. However, puberty initiates growth plate closure and, after puberty, most bone growth ceases. In many species, sex and age affect the relationship between bone strength and liveweight. The aim of this study was to examine how bone strength in two different limb bones was affected by age and sex in beef-cross-dairy cattle. The metacarpus and humerus was collected at time of slaughter and scanned using a peripheral quantitative computed tomography (pQCT) scanner to obtain measures of bone size and morphology from three cohorts of beef-cross-dairy cattle. Age, sex and live weight were also recorded. Live weight was the main predictor of bone size and strength, but age and sex influenced this relationship. This was reflected in heifers having a metacarpus that was shorter with less cross-sectional area and bone content than steers of the same liveweight and limited differences between steers and bulls of the same liveweight. At the same bone size older animals were heavier demonstrating an effect of age on bone maturity.

**Abstract:**

In cattle, limited data have been reported about the relationship between live weight, bone size, and strength and how this relationship can be altered by factors such as sex and age. The aim of this study was to describe the relationship of peripheral quantitative computed tomography (pQCT)-derived parameters of bone strength and morphology with live weight, age and sex in beef-cross-dairy cattle. All animals were weighed the day before slaughter. The metacarpus and humerus were collected at slaughter and scanned at the mid-diaphysis using pQCT. Live weight was the primary explanatory variable for bone size and strength in all cohorts. However, the effect of age was significant, such that magnitude of response to liveweight was less in the 24-month-old cohort. Sex was significant within cohorts in that bulls had a shorter metacarpus than steers and heifers had a shorter metacarpus than steers at age of slaughter.

## 1. Introduction

As live weight increases, bone must increase in size and strength to maintain strain within physiological limits [1]. The application of this theory has been supported by research looking in to the relationship between bone and strain where increases in live weight or loading result in a rise in bone strength [2,3]. The nature and magnitude of the bone response is dependent on the region within the bone and the stage of maturation of the animal. The mid-diaphyseal site of long bones must resist the bending and torsion arising from locomotion, so increases in live weight and therefore loading forces must result in an increase in strength [2]. The primary response in long bones to increase “bone strength” is increasing bone size via appositional growth. During the bone remodelling process, bone is removed from the inner endosteal side and deposited on the outer periosteum to provide a larger cross-sectional area with a minimal increase in cortical bone thickness [4]. However, the ability for bone to increase strength via appositional growth is limited once the animal attains puberty and there is a reduction in longitudinal growth when the growth plate is closed at bone maturity [5].

An important cue for the initiation of bone maturity is the increase in oestrogen at puberty in both females and males [6]. In cattle, the common definition of puberty is the onset of regular oestrous cycles for heifers, and the production of viable semen for bulls, both indicative of the animal developing the maturity to reproduce [7,8]. For cattle, puberty is attained when animals reach approximately 48–51% of their mature body weight [9]. At the onset of puberty, oestrogen is secreted from the ovaries in heifers and the Leydig cells in the testes of bulls in low concentrations. In the early stages of puberty, the increase in sex and growth hormones causes elongation of long bones, colloquially referred to as a “growth spurt” [6]. Distal portions of the limbs such as the metacarpus have limited longitudinal growth potential compared to proximal bones such as the humerus [10]. In late puberty, higher concentrations of oestrogen inhibit bone resorption by reducing the number of osteoclasts produced and limiting osteocyte apoptosis [5]. A decrease in bone growth promotes the closure of the physis and allows for endosteal and trabecular bone formation. A lack of oestrogen, i.e., castration will prevent puberty acquisition and delay bone maturity resulting in a longer bone [11,12].

Inadequate bone growth prior to puberty and bone maturity can result in a smaller bone size and limited bone strength. A potential cause for reduced bone size is a period of malnutrition, where bone growth ceases and the physis is temporarily sealed. When bone growth resumes, the seal on the physis is displaced and is observed histologically and on radiographs as a growth arrest line (Harris line) [13].

Spontaneous humeral fractures have been reported in first calving dairy cows in New Zealand [14]. It is estimated that 4% of dairy farms are affected by humeral fractures a year with an on-farm prevalence of 2–25% of replacement heifers resulting in a significant welfare issue. Due to the location of the humerus, the ability to examine the bone is restricted to post-mortem, however, bones such as the metacarpus are easily accessible and have been shown to be a good predictor of bone size and strength in the humerus [15]. Humeri from heifers with humeral fractures often have a reduced periosteal circumference and bone volume to total bone volume ratio (BV/TV). Growth arrest lines were also present indicating a period of malnutrition, suggesting this may predispose affected animals to fracture [14]. This highlights the importance of understanding bone growth prior to puberty. Therefore, the aim of this study was to examine the effect of sex and age on the relationship between live weight and pQCT derived measures of bone mass and strength in the metacarpus and humerus of cattle.

## 2. Materials and Methods

### 2.1. Animals

The experiment included three cohorts of beef-cross-dairy breed calves reared at pasture on three commercial farms in the Manawatu region of New Zealand. The first cohort were Stabilizer-sired calves born to 2-year-old, Friesian–Jersey crossbred heifers. Cohort two consisted of Hereford-sired, male calves born to Friesian and Friesian–Jersey crossbred dairy cows, and cohort three consisted of Hereford-sired, steers born to Friesian and Friesian–Jersey crossbred dairy cows.

Each cohort included a control group (steers slaughtered between 8–12 months of age) and a comparison group. At slaughter, measures of bodyweight, stature and, bone mass and architecture, were subsequently compared between the young steers and the comparison group of young heifers, young bulls or older steers at farms 1–3, respectively. The calves were purchased and moved to their respective farm at approximately 100 kg live weight, after having been artificially reared using commercial calf-rearing systems that involved weaning off milk at around 70 kg, and feeding high protein meal to approximately 100 kg live weight (around 3 months of age) [16]. Cattle were grazed under commercial conditions and managed with the aim of achieving high growth rates (target 1 kg/d). The predominant forage on offer was perennial ryegrass (*Lolium perenne*) and white clover (*Trifolium repens*) pasture. To achieve target growth rates during periods of low pasture growth, forage crops (plantain and chicory) were used to supplement the base pasture diet. Within cohort, all animals were grazed together as a single mob.

#### 2.1.1. Cohort One (Steers (*n* = 14) Versus Bulls (*n* = 19)

Cohort one consisted of Hereford-sired, male calves born to Friesian and Friesian–Jersey crossbred dairy cows. Fourteen randomly selected calves had been castrated before 4 weeks of age, whilst the remaining 19 were left entire. At 11 months of age the calves were processed at a commercial abattoir. All procedures related to the management and use of these animals during the experiment were approved by the Massey University Animals Ethics Committee (MUAEC 18/120).

#### 2.1.2. Cohort Two (Steers (*n* = 12) Versus Heifers (*n* = 12))

The initial group consisted of 140 stabilizer-sired calves born to 2-year-old, Friesian–Jersey crossbred heifers. At 11 months of age, the 30 heaviest heifers and 30 heaviest steers were identified, and 12 of each sex were randomly selected from these subsets for processing at a commercial abattoir. All procedures related to the management and use of the animals during the experiment were approved by the Massey University Animals Ethics Committee (MUAEC 18/120).

#### 2.1.3. Cohort Three (Steers Aged 8–12 Months (*n* = 60) or 24 Months (*n* = 20))

A cohort of 80 Hereford-sired weaner steers from predominantly Friesian–Jersey crossbred dams were purchased from a single commercial calf rearer at approximately 100 kg (3 months of age). Calves were managed as a single group and randomly assigned at 8 months of age to one of four slaughter ages, being 8, 10, 12 or 24 months of age with each treatment group balanced for live weight at 8 months of age [17]. Cattle were processed at a commercial abattoir. All procedures related to the management and use of these animals during the experiment were approved by the Massey University Animals Ethics Committee (MUAEC 17/73).

### 2.2. Sample Collection

Unfasted live weight was recorded on farm the day before slaughter. Cattle underwent commercial slaughter and standard dressing procedures. The left metacarpus was collected at time of slaughter and the humerus was collected the following day at the boning-out stage. Both the humerus and metacarpus were double wrapped in plastic film and stored at −20 °C until scanning.

### 2.3. Scanning

The pQCT scanning was carried out using the same protocol previously reported by Gibson et al. [3]. Briefly, pQCT scanning was carried out using an XCT 2000 peripheral quantitative computed tomography machine (Stratec Medical, Pforzheim, Germany). For each bone, a 2 mm slice at the mid diaphysis was obtained with a voxel size of 0.3 mm. In the metacarpus, the mid-diaphysis was defined as 50% of the total bone length using the lateral aspect of the lateral condyle of the MC4 and the proximal aspect of the lateral MC4. In the humerus, the mid-diaphysis was defined as 50% of total bone length, measured from the proximal end of the humeral head at the lateral aspect, to the end of the trochlea at the distal end.

Data derived from the scan included measures of total bone content, cortical (>710 mg/mL CaHA) and subcortical content, cortical and subcortical density, trabecular content, trabecular density, total area, trabecular area, cortical and subcortical area, cortical content, cortical density, cortical area, cortical thickness, periosteal circumference, endosteal circumference and stress–strain index (SSI). The stress–strain index is a derived measure of bone strength using pQCT data and avoids the requirement for destructive testing using techniques such as the three-point bending test. The stress–strain index described the ability of bone to withstand bending from lateral, dorso-palmar and torsional forces and is calculated by incorporating the index of material stiffness (bone mineral density) and bone geometry (cross-sectional moment of inertia) [18]. The pQCT variables will be referred to as bone parameters in the statistical models.

### 2.4. Statistical Analysis

Statistical analysis was conducted using the Statistical Analysis System software version 9.4 (SAS institute Inc., Carey, NC, USA). All analyses were conducted using general linear models. The models for live weight and bone length included the fixed effect group (control vs. comparison).

#### 2.4.1. Sex Effect

Bone parameters were compared between groups for each cohort. Models included the fixed effect of group (control vs. comparison) and the covariate of live weight. The interaction of group and live weight was considered but removed if not significant (*p* > 0.05).

#### 2.4.2. Age Effect

Live weight was compared between groups for cohort three. Models included the fixed effect of group (8–12 months vs. 24 months) and the covariate of bone parameter. The interaction of group and bone parameter was considered but removed if not significant (*p* > 0.05).

## 3. Results

### 3.1. Effect of Sex/Cohort One and Two

There was limited effect of sex on bone parameters between bulls and control steers from cohort one in both the metacarpus and humerus when liveweight was included in the model (Table 1). Sex had a significant effect on metacarpus length. Most bone parameters had a moderate association with live weight (R^2^ = 0.28−0.65) with the exception of endosteal circumference in the metacarpus and endosteal circumference and cortical bone density in the humerus which had limited variation which was reflected in the lower R^2^ values for model fit.

In the humerus, cortical bone thickness was greater (*p* < 0.001) in steers than bulls (8.4 vs. 8.3 mm, respectively). There was a weight by sex interaction for cortical bone thickness (*p* = 0.045), such that cortical bone thickness increased with live weight at a rate of 0.01 mm/kg in steers and 0.02 mm/kg in bulls.

In cohort two heifers had a shorter bone length and lower stress–strain index in the metacarpus than steers at the same live weight (Table 2). Similarly, heifers had lower total bone content, cortical bone area and stress–strain index in the humerus than steers of the same live weight. Live weight had a significant effect for most parameters in both bones with the exceptions being cortical bone thickness in the metacarpal and humerus, and endosteal circumference in the humerus. Most bone parameters had a moderate association with live weight (R^2^ = 0.28−0.62). There was limited variation of cortical bone thickness in the metacarpus and bone length and endosteal circumference in the humerus, which was reflected in the lower R^2^ values for model fit.

### 3.2. Effect of Age/Cohort Three

There was an effect of age on the relationship of live weight with all pQCT measured bone parameters. Live weight was greater at the same bone parameter value for the older animals. There was no relationship of live weight with cortical density in the metacarpus and humerus and also endosteal circumference in the humerus (Table 3). All bone parameters had a strong association with live weight (R^2^ = 0.90−0.97) in both the metacarpus and humerus.

In the metacarpus, there was a significant interaction of bone parameter and group in total bone area, total bone content, cortical bone thickness, cortical bone area, cortical bone content and the stress–strain index. Therefore, younger animals had a larger gain in live weight per unit of bone parameter (Table 4).

## 4. Discussion

The results in the present study agree with an earlier observation from a younger cohort of cattle that identified live weight as the major explanatory variable for measures of bone mass and strength in the metacarpus and humerus [3]. In addition, in the current study, it was demonstrated that the relationship of live weight with measures of bone mass and strength was modified by age and sex.

Bone minimises strain such as that generated by increased bodyweight by changing content and geometry (size) [1,19]. However, as the animal reaches mature height and longitudinal bone growth ceases the expectation is there will be a decrease in the magnitude of the bone response to increasing live weight. This was observed in the age interaction in the metacarpus with the 24-month-old cohort demonstrating a two to three-fold reduction in the bone response for each kg increase in liveweight. Cattle are not cursorial animals and thus most locomotion is at low velocities (strain rate) [20]. Within commercial farming systems even management on hill properties did not appear to provide sufficient strain to induce an increase in bone strength [21]. In contrast, bone parameters in the humerus did not have an age interaction, which implies that the humerus was still capable of growth at 24 months in these cattle. This finding agrees with the pattern of bone maturity described by where bones in the distal limb in the cow, such as the metacarpus, mature earlier than bones in proximal limb, such as the humerus. Differences in bone response to increases in liveweight is also a result of differences in the anatomical location of the bone and therefore differences in the direction and magnitude of the mechanical forces. The larger stress–strain index in the humerus demonstrates that at the mid-diaphysis, the humerus is subjected to a much larger force than in the metacarpus. The metacarpus undergoes compressional forces from stance resulting in a low strain rate [22]. Whereas in the humerus, torsional forces are placed on bone via surrounding muscles during locomotion [23].

Steers had a longer metacarpus than bulls and heifers but had similar humeral lengths. Therefore, bone maturation (closure of the physis) in the distal limb of heifers and bulls had begun, but not in the steers. A likely cause for this difference in bone maturity is that the hormonal cue for the cessation of longitudinal bone growth, via puberty acquisition, was not occurring for steers. During puberty, increases in circulatory concentration of both oestrogen (in heifers and bulls) and testosterone (in bulls) are associated with physeal closure [5]. However, each sex hormone has a differing subsequent effect on bone mass as the animal ages. At the onset of puberty oestrogen inhibits further appositional growth thus effectively restricting the periosteal circumference to that achieved at the time of puberty [6]. Conversely, testosterone has a limited effect on bone resorption and does not inhibit increases in periosteal circumference [24,25]. The effect of testosterone results in a larger bone size in males and a later bone maturity than in the female [10].

The presence of high concentrations of testosterone in bulls promotes increases in muscularity compared to heifers with low concentrations of testosterone [26]. Increased muscle mass is associated with increased bending strain on the long bones and therefore increases in bone mass [19]. Muscle and bone form the bone-muscle unit to provide a signalling pathway between each other. Skeletal muscle secretes growth factors such as insulin like growth factor (IGF-I) which stimulates bone growth [27]. Therefore, increases in muscularity, due to increased circulatory concentrations of testosterone in bulls, should promote increases in the cross-sectional size of bone by appositional growth to prepare for a greater bending strain. The lack of testes in steers prevents testosterone from being produced but, the genetics for a large mature size will create a larger frame size than a heifer without the muscularity of a bull to promote the larger cross-sectional area in long bones [26,28]. The greater periosteal circumference and endosteal circumference contributed to the greater total bone content observed in the steers than the heifers and reflects the effect of oestrogen inhibiting appositional growth in heifers. The lack of difference in periosteal circumference, endosteal circumference and total bone content between bulls and steers indicates that appositional bone growth in both the metacarpus and humerus was still occurring at 11 months of age possibly due to a lack of a pubertal spike in circulatory testosterone and oestrogen. Therefore, to quantify differences between steers and bulls, due to the presence and absence of testosterone, the animals would need to be older and post-pubertal.

With an estimated mature weight of 770 kg, bulls in the current study would have been approximately 39.7% of mature weight, therefore, it is likely that bulls of this age were pre-pubertal [29]. Therefore, longitudinal growth of the long bones, particularly the humerus, would continue after the age and weight at which the steers and bulls were slaughtered. As bulls reached puberty it would be expected that the increase in oestrogen will slow bone growth and mineralisation will increase. The effect of this would then be the divergence of the bone parameters, and the primary mechanisms used to increase resistance to bending strain, between the bulls and steers. This post-pubertal divergence in bone parameters between steers and bulls has been reported for the femur in cattle [12]. Post puberty, bulls had shorter femurs than steers of the same live weight. However, the weight of the femur remained the same, possibly due to greater total bone density in the femur of the bulls. However, using live weight as a predictor for puberty does not precisely identify when puberty attainment occurred. The use of hormone assay would have been an effective way to identify puberty status but was unable to be undertaken due to the opportunistic nature of the bone collection from another study.

Increases in SSI in both the metacarpus and humerus were achieved by increasing the cross-sectional area of the mid-diaphysis, which reflects a common mammalian response to growth and loading in the long bones of the limb [2]. In first lactation dairy heifers, heifers may present with humeral fractures, which are associated with reduced periosteal circumference and reduced BV/TV compared with control heifers [14]. The data from the present study indicate that at 24 months the humerus was still experiencing appositional and longitudinal growth and is sensitive to changes in live weight. Therefore, in cattle, appositional growth of the humerus may be sensitive to periods of reduced nutrition between puberty and first lactation. Industry growth data provide evidence that many dairy heifers may encounter a “growth check”(reduced live weight gain) at this time [30]. The resultant reduction in appositional growth of the humerus (and hence reduced bone mass and resistance to bending), due to the “growth check”, may predispose dairy heifers to humeral fractures due to an inability to cope with mobilisation of bone tissue from the endosteal surface to supply calcium during the first lactation.

## 5. Conclusions

Live weight was the major explanatory variable of bone size and strength, with age and sex modifying the relationship. Differences in bone maturity meant that the rate of response in the metacarpus at 24 months to increases in live weight were less than that observed in 8–12 months. However, there was no interaction of age and bone response in the humerus, indicating it was still capable of growth.

The differences in growth capacity of the metacarpus and humerus was reflected in the sexassociated differences between groups, with the presence of oestrogen inhibiting longitudinal growth in the metacarpus and lack of sex hormones in the steers delaying inhibition of longitudinal growth. Heifers were characterised by shorter and smaller long bones in the metacarpus. However, the effect of puberty on the humerus had not yet occurred with minimal signs of bone maturation, indicating, that proximal bone growth was still occurring at 11 months.

Increased SSI with live weight and age occurred by increasing the cross-sectional area in the mid diaphysis rather than increasing bone density. At 24 months the humerus was still highly responsive to changes in live weight and thus any “growth check” would inhibit the ability to provide adequate cross-sectional area. Failure to attain optimal cross-sectional area at the time of peak lactation and associated increase in bone mobilisation could predispose heifers to humerus fracture.

## Figures and Tables

**Table 1:** Least squared means and standard error for bone parameters in the metacarpus and humerus with the effect of sex in bulls and steers at 11 months of age from cohort one where there was no interaction of weight and sex.

	Bull	Steer	Live Weight Coefficient	*p*-Value		R^2^
				Live Weight	Sex	
Live weight (kg)	305.9 ± 7.1	303.0 ± 6.9			0.773	0.00
**Metacarpus**						
*n*	16	17				
Bone length (mm)	205.8 ± 1.4	211.3 ± 1.3	0.21 ± 0.03	<0.001	0.008	0.59
Periosteal Circumference (mm)	98.3 ± 0.7	97.9 ± 0.7	0.10 ± 0.02	<0.001	0.661	0.50
Endosteal circumference (mm)	61.2 ± 1.0	61.3 ± 0.9	0.05 ± 0.02	0.055	0.897	0.12
Total bone area (mm^2^)	770.5 ± 11.2	763.0 ± 10.8	1.5 ± 0.3	<0.001	0.634	0.50
Total bone content (mg/mm)	607.6 ± 8.0	600.7 ± 7.8	1.5 ± 0.2	<0.001	0.573	0.64
Cortical bone thickness (mm)	5.9 ± 0.1	5.8 ± 0.1	0.008 ± 0.002	0.002	0.457	0.28
Cortical bone area (mm^2^)	471.4 ± 6.9	462.4 ± 6.7	1.1 ± 0.2	<0.001	0.355	0.56
Cortical bone density (mg/cm^3^)	1206 ± 3	1213 ± 3	0.3 ± 0.1	<0.001	0.083	0.43
Cortical bone content (mg/mm)	568.6 ± 8.2	560.9 ± 8.0	1.4 ± 0.2	<0.001	0.505	0.61
Stress–strain index (mm^3^)	4205 ± 84	4111 ± 81	13.6 ± 2.1	<0.001	0.426	0.59
**Humerus**						
*n*	16	17				
Bone length (mm)	233.5 ± 1.7	235.1 ± 1.6	0.14 ± 0.04	0.002	0.497	0.28
Periosteal Circumference (mm)	132.8 ± 1.2	133.3 ± 1.2	0.18 ± 0.03	<0.001	0.784	0.52
Endosteal circumference (mm)	80.6 ± 1.4	80.4 ± 1.4	0.10 ± 0.03	0.012	0.930	0.19
Total bone area (mm^2^)	1407 ± 26	1417 ± 25	3.7 ± 0.7	0.001	0.804	0.51
Total bone content (mg/mm)	1171 ± 18	1184 ± 17	3.3 ± 0.4	<0.001	0.604	0.65
Cortical bone area (mm^2^)	888.5 ± 14.1	899.2 ± 13.6	2.5 ± 0.4	<0.001	0.589	0.62
Cortical bone density (mg/cm^3^)	1247 ± 4	1243 ± 4	−0.1 ± 0.1	0.315	0.436	0.05
Cortical bone content (mg/mm)	1108 ± 16	1118 ± 16	3.0 ± 0.4	<0.001	0.672	0.64
Stress–strain index (mm^3^)	11,352 ± 268	11,503 ± 2560	46.4 ± 6.8	<0.001	0.688	0.61

**Table 2:** Least square means and standard error of bone parameters adjusted for live weight of heifers and steers at 11 months of age from cohort two where there was no interaction of weight and sex.

	Heifer	Steer	Live Weight Coefficient	*p*-Value		R^2^
				Weight	Sex	
Live weight (kg)	314.3 ± 5.2	323.4 ± 5.2			0.228	0.07
**Metacarpus**						
*n*	12	12				
Bone length (mm)	198.5 ± 1.9	204.3 ± 1.9	0.1 ± 0.1	0.140	0.046	0.30
Periosteal Circumference (mm)	91.6 ± 1.0	94.7 ± 1.0	0.16 ± 0.04	0.001	0.054	0.53
Endosteal circumference (mm)	54.2 ± 1.4	57.3 ± 1.4	0.1 ± 0.1	0.015	0.145	0.37
Total bone area (mm^2^)	670.1 ± 15.6	714.9 ± 15.6	2.3 ± 0.6	0.001	0.059	0.52
Total bone content (mg/mm)	556.3 ± 9.2	582.9 ± 9.2	1.2 ± 0.4	0.005	0.058	0.46
Cortical bone thickness (mm)	6.0 ± 0.1	6.0 ± 0.1	0.001 ± 0.005	0.817	0.999	0.00
Cortical bone area (mm^2^)	433.4 ± 7.5	451.6 ± 7.5	1.0 ± 0.3	0.003	0.105	0.47
Cortical bone density (mg/cm^3^)	1210 ± 4	1213 ± 4	−0.5 ± 0.2	0.005	0.681	0.32
Cortical bone content (mg/mm)	524 ± 9	548 ± 9	1.0 ± 0.4	0.012	0.080	0.41
Stress–strain index (mm^3^)	3529 ± 102	3854 ± 102	14.5 ± 4.1	0.002	0.038	0.52
**Humerus**						
*n*	12	12				
Bone length (mm)	233.2 ± 2.5	235.3 ± 2.6	0.1 ± 0.1	0.279	0.584	0.10
Periosteal Circumference (mm)	125.9 ± 1.4	128.8 ± 1.5	0.2 ± 0.1	0.004	0.178	0.46
Endosteal circumference (mm)	75.4 ± 1.9	75.2 ± 2.0	0.1 ± 0.1	0.195	0.950	0.09
Total bone area (mm^2^)	1265 ± 28	1322 ± 30	3.8 ± 1.2	0.004	0.192	0.45
Total bone content (mg/mm)	1059 ± 19	1149 ± 20	2.8 ± 0.8	0.002	0.005	0.62
Cortical bone thickness (mm)	8.0 ± 0.2	8.5 ± 0.2	0.01 ± 0.01	0.073	0.072	0.34
Cortical bone area (mm^2^)	808.0 ± 16.2	869.3 ± 16.9	2.5 ± 0.7	0.001	0.019	0.60
Cortical bone density (mg/cm^3^)	1245 ± 5	1253 ± 6	−0.6 ± 0.2	0.013	0.300	0.27
Cortical bone content (mg/mm)	1004 ± 18	1089 ± 19	2.7 ± 0.7	0.002	0.005	0.61
Stress–strain index (mm^3^)	9447 ± 270	10,564 ± 283	39.6 ± 11.0	0.002	0.011	0.59

**Table 3:** Live weight adjusted for each bone parameter in the metacarpus and humerus of control steers (8–12 months) and 24-month-old steers from cohort three for which there was no interaction of bone parameter and group. Values are the least squared mean (±standard error) live weight for each age group adjusted to the overall mean value of the bone parameter identified in that row.

	8–12 Months Live Weight	24 Months Live Weight	Bone Parameter Coefficient	*p*-Value		R^2^
				Bone Parameter	Group	
**Metacarpus**						
*n*	57	22				
Live weight (kg)	301.1 ± 5.7	580.1 ± 9.2			<0.001	0.90
Bone length (mm)	305.5 ± 5.8	568.2 ± 10.0	1.0 ± 0.4	0.016	<0.001	0.90
Periosteal Circumference (mm)	328.4 ± 6.4	509.5 ± 13.5	5.1 ± 0.8	<0.001	<0.001	0.93
Endosteal circumference (mm)	304.4 ± 5.8	571.6 ± 9.8	2.0 ± 0.9	0.034	<0.001	0.90
Cortical bone density (mg/cm^3^)	298.4 ± 6.2	587.1 ± 11.1	−0.4 ± 0.4	0.273	<0.001	0.90
**Humerus**						
*n*	17	18				
Live weight (kg)	345.8 ± 6.4	568.8 ± 6.2			<0.001	0.95
Bone length (mm)	372.7 ± 7.4	543.3 ± 7.1	2.1 ± 0.4	<0.001	<0.001	0.97
Periosteal Circumference (mm)	374.9 ± 9.4	541.3 ± 9.1	2.2 ± 0.6	<0.001	<0.001	0.97
Endosteal circumference (mm)	356.7 ± 8.3	558.4 ± 8.0	1.1 ± 0.6	0.059	<0.001	0.95
Total bone area (mm^2^)	374.9 ± 9.1	541.3 ± 8.7	0.09 ± 0.02	<0.001	<0.001	0.97
Total bone content (mg/mm)	379.7 ± 9.5	536.7 ± 9.1	0.15 ± 0.03	<0.001	<0.001	0.97
Cortical bone thickness (mm)	351.7 ± 7.2	563.2 ± 7.0	11.5 ± 7.0	<0.110	<0.001	0.95
Cortical bone area (mm^2^)	377.4 ± 9.1	538.9 ± 8.7	0.19 ± 0.04	<0.001	<0.001	0.97
Cortical bone density (mg/cm^3^)	344.3 ± 6.6	570.2 ± 6.4	−0.3 ± 0.3	0.377	<0.001	0.95
Cortical bone content (mg/mm)	377.8 ± 9.5	538.6 ± 9.1	0.14 ± 0.04	<0.001	<0.001	0.97
Stress–strain index (mm^3^)	371.1 ± 8.7	544.9 ± 8.3	0.007 ± 0.002	<0.001	<0.001	0.96

**Table 4:** Least squared means and standard error for live weight adjusted for each bone parameter in the metacarpus of control steers (8–12 months) and 24-month-old steers from cohort three. Regression coefficient for each group is presented with the interaction of bone parameter and group being significant.

			Coefficient		*p*-Values			
	8–12 Months Live Weight	24 Months Live Weight	8–12 Months	24 Month	Bone Parameter	Group	Bone Parameter x Group Interaction	R^2^
Total bone area (mm^2^)	334.9 ± 7.1	544.3 ± 21.9	0.4	0.2	<0.001	<0.001	0.042	0.93
Total bone content (mg/mm)	336.7 ± 0.4	515.1 ± 24.8	0.7	0.3	<0.001	<0.001	<0.001	0.96
Cortical thickness (mm)	341.5 ± 7.5	541.2 ± 29.7	67.9	25.3	<0.001	0.004	0.048	0.94
Cortical bone area (mm^2^)	365.0 ± 7.0	516.6 ± 24.4	0.9	0.3	<0.001	<0.001	<0.001	0.96
Cortical content (mg/mm)	368.3 ± 7.5	513.1 ± 25.3	0.7	0.3	<0.001	<0.001	<0.001	0.96
Stress–strain index (mm^3^)	351.2 ± 7.9	542.0 ± 22.1	0.06	0.02	<0.001	<0.001	<0.001	0.94

## Data Availability

Data will be available from the first author upon request.
